# Circulating monocyte subsets and heart failure prognosis

**DOI:** 10.1371/journal.pone.0204074

**Published:** 2018-09-21

**Authors:** Elena Elchinova, Iris Teubel, Santiago Roura, Marco A. Fernández, Josep Lupón, Carolina Gálvez-Montón, Marta de Antonio, Pedro Moliner, Mar Domingo, Elisabet Zamora, Julio Núñez, Germán Cediel, Antoni Bayés-Genís

**Affiliations:** 1 Heart Failure Unit and Cardiology Service, Germans Trias i Pujol University Hospital, Badalona, Spain; 2 Department of Medicine, Universitat Autònoma de Barcelona, Barcelona, Spain; 3 Inselspital, University Hospital, Bern, Switzerland; 4 Flow Cytometry Facility, Germans Trias i Pujol Health Science Research Institute, Badalona, Spain; 5 CIBERCV, Instituto de Salud Carlos III, Madrid, Spain; 6 ICREC Research Program, Germans Trias i Pujol Health Science Research Institute, Badalona, Spain; 7 Cardiology Department, Hospital Clínico Universitario, INCLIVA, Universitat de València, Valencia, Spain; Institut Cochin, FRANCE

## Abstract

Monocytes are a heterogeneous population of effector cells with key roles in tissue integrity restoration and maintenance. Here, we explore the association of monocyte subsets and prognosis in patients with ambulatory heart failure (HF). Monocyte subsets were classified as classical (CD14^++^/CD16^–^), intermediate (CD14^++^/CD16^+^), or non-classical (CD14^+^/CD16^++^). Percentage distribution and absolute cell count were assessed in each subset, and multivariable Cox regression analyses were performed with all-cause death, HF-related hospitalization, and the composite end-point of both as dependent variables. 400 patients were consecutively included (72.8% male, age 69.4±12.2 years, 45.5% from ischemic aetiology, left ventricle ejection fraction (LVEF) 41.6% ±14.5, New York Heart Association (NYHA) class II 62.8% and III 30.8%). During a mean follow-up of 2.6±0.9 years, 107 patients died, 99 had a HF-related hospitalization and 160 suffered the composite end-point of all-cause death or HF-related hospitalization. Monocyte subsets assessed in percentages were not independently associated to any of the end-points. When considering number of cells/μL, intermediate subset was independently associated with an increase of all-cause death (HR 1.25 [95% CI 1,02–1.52], p = 0.03), and the composite end-point HR 1.20 [95% CI 1,03–1.40], p = 0.02). The presented findings show that absolute cell count of monocyte subsets was preferred over monocyte percentage for prognosis stratification for outpatients with HF. The intermediate monocyte subset provides information on increased risk of all-cause death and the composite end-point.

## Introduction

Heart failure (HF) is a syndromic disease associated with significant economic burden and clinical manifestations [1,2]. Morbidity and mortality continues to be unacceptably high for patients with HF, despite significant research and medical progress. A better risk stratification by an improved understanding of the underlying pathogenic mechanisms and potentially valuable prognostic markers could be a key for the optimal determination of patients who would benefit from close follow-up and more aggressive treatment. However, established prognostic factors including the New York Heart Association (NYHA) functional classification, the left ventricle ejection fraction (LVEF), age, sex, aetiology, comorbidities and laboratory markers all fail to completely and individually predict disease progression and mortality [3–6]. Risk stratification could be improved by the incorporation of biomarkers associated with different pathophysiological pathways, not reflected by established mortality risk factors. The search for biomarkers for HF diagnosis and prognosis has become a major research focus over the last decade [6–8].

The significance and importance of monocyte count and subset distribution in HF is unknown, and their ability to act as prediction factors for the severity and progression of the disease is not yet well established [9,10], although previous reports have indicated an association between monocyte subpopulations and cardiovascular events in different pathologies [11–14]. Monocytes and their macrophage derivates play a crucial role in the immune system, and participate in host defense, immunoregulation, and tissue repair. In particular, there are three distinct subsets of monocytes, defined as classical (CD14^++^/CD16^–^), intermediate (CD14^++^/CD16^+^) or non-classical (CD14^+^/CD16^++^) by Ziegler-Heitbrock and colleagues among others [15], which can be differentially detected by multicolor flow cytometry analysis [16].

In the present study, we investigate the clinical relevance of monocyte count and distribution in patients with ambulatory HF, and the value of monocyte subsets to establish disease prognosis.

## Materials and methods

### Study population

Ambulatory patients consecutively treated at a multidisciplinary HF Clinic from December 2013 to April 2015 were included in this study, independently of the data of their entry into the HF Clinic program. The referral inclusion criteria have been described elsewhere [17,18]. All study procedures were performed in accordance with the ethical standards outlined in the Helsinki Declaration of 1975 as revised in 2013 [19], this study was approved by the local ethics committee (Comitè d’Ètica de la Investigació, Hospital Universitari Germans Trias i Pujol, Ref. CEI: PI-13-057), and all participants provided written informed consent.

### Follow-up and outcomes

All patients made follow-up visits at regular, predefined intervals, and made additional visits as required in cases of decompensation [17,18]. The schedule included a minimum of quarterly visits with nurses, biannual visits with physicians, and elective visits with geriatricians, psychiatrists, nephrologists, and rehabilitation physicians. Patients were contacted by telephone if they did not attend a regular visit.

The primary end-points were all-cause death and the composite of all-cause death or HF-related hospitalization. Fatal events were identified from electronic clinical records and by contact with the patients’ relatives if necessary. When needed, data were verified by comparison with records stored in the databases of the Catalan and Spanish health systems. Events were adjudicated by two of the authors (EE and JL) and three clinical and research nurses (BG, JG and JR). Follow-up was closed in May 2017.

### Blood extraction and flow cytometry analysis

Peripheral blood samples (~3 mL) were collected into EDTA tubes via standard forearm venipuncture performed between 9:00 am and 11:00 am, and processed within 4 h post-collection. Samples and data from patients included in this study were processed and collected by the IGTP-HUGTP Biobank integrated in the Spanish National Biobanks Network of Instituto de Salud Carlos III (PT13/0010/0009) and Tumour Bank Network of Catalonia.

In brief, 100 μL of fresh peripheral blood (tube A) was stained with titrated amounts of anti-human CD86-BV605, CD14-BV785, and CD16-BV421 antibodies (Biolegend, San Diego, CA) during 15 min in the dark at room temperature. Red blood cells were then lysed following 10-minute incubation with 2 mL PharmLyse solution (BD Biosciences, San Diego, CA), and the resulting cell suspension was washed twice with phosphate-buffered saline. For monocyte absolute cell counts, 50 μL of unprocessed blood from the same donor were also stained (tube B) with anti-human CD86-PE (BD Biosciences) antibody and 50 μL Perfect-Count beads (concentration ranging from 1,056 to 1,067 beads/μL, depending on batch; Cytognos, Salamanca, Spain) were also added as reference counting beads in a lyse no-wash method. Fluorescence minus one controls were used to determine positive and negative staining boundaries for CD14 and CD16 antigens.

Monocyte subsets were classified as CD14^++^/CD16^–^ (classical), CD14^++^/CD16^+^ (intermediate), and CD14^+^/CD16^++^ (non-classical). Frequency of each monocyte subset regarding total monocyte population (CD86^+^) were obtained from tube A and absolute counts (expressed as number of cells/μL) were calculated by multiplying the percentage of each subset by the total monocyte population (CD86^+^) count performed from tube B. All samples were acquired in the next hour after staining protocol on a Fortessa SORP flow cytometer (BD Biosciences) equipped with four lasers (100-mW 488 nm, 150mW 532 nm, 50mW 405 nm, and 100mW 640 nm) by using the sample acquisition and analysis FACSDiva v6.2 (BD Biosciences) and FlowJo vX (Tree Star Inc., Ashland, OR) software, respectively. We performed routine daily quality control tests with Cytometer Setup & Tracking Beads (BD Biosciences) in accordance with the manufacturer’ s instructions. The optimal voltage ranges and linearity for photomultipliers were selected using 6-peak Rainbow Calibration Particles and Unstained Comp-Beads (BD Biosciences). All laboratory measurements were performed by staff blind to the clinical characteristics.

### Statistical analysis

Categorical variables are expressed as percentages, and continuous variables are expressed as means ± standard deviation (SD) or medians (25th–75th percentiles) for normal and non-normal distributions, respectively. Data distributions were assessed with normal Q–Q plots.

Differences between groups were assessed with chi-squared test, Student’s t-test and Mann-Whitney U test as appropriate, both for monocyte subset percentage and concentration. Correlations were assessed with Pearson test or Spearman Rho test as appropriate. Univariable Cox regression analyses were performed with all-cause death, HF-related hospitalization, and the composite end-point of both as the dependent variables and age, sex, ischemic etiology, New York Heart Association (NYHA) functional class, left ventricular ejection fraction (LVEF), hemoglobin, sodium, estimated glomerular filtration rate, NTproBNP (Log-transformed and per 1 SD), and monocyte subset percentages and absolute cell count (Log-transformed and per 1 SD), as independent variables. Multivariable analyses were performed with the same dependent variables and the subsets of monocytes found to be statistically significant in the univariable analysis as independent variables, together with variables with p<0.1 in the univariable analysis and sex (considered clinically relevant) as co-variables. Colinearity between monocyte subsets was discarded by variance inflation factor (VIF). We adopted the Gray method of including competing risk for the analyses of HF-related hospital admission, considering death as the competing event for HF-related hospitalizations. Survival curves for all-cause death and the composite end-point, were plotted based on quartiles of intermediate monocyte subset absolute cell count. Statistical analyses were performed with SPSS 15 (SPSS Inc., Chicago, IL) and STATA V.13.0 (StataCorp LLC, College Station, TX,), and a two-sided p<0.05 was considered significant.

## Results

400 patients were consecutively included in the study. The demographic and clinical characteristics of the patients at the data of sample collection relative to all-cause death are listed in Table 1. Briefly, enrolled patients were middle aged, predominantly males with ischemic systolic heart failure, typically NYHA functional class II or III (2 patients were NYHA class IV), with long-term HF duration, moderately depressed LVEF, and treated according to guideline-derived recommendations. During a mean follow-up of 2.6 ± 0.9 years, 107 patients died, 99 had a HF-related hospitalization and 160 suffered the composite end-point of all-cause death or HF-related hospitalization.

**Table 1:** Baseline demographic, clinical and biochemical data of the study participants.

	Total	Alive	Deceased	p-value
	n = 400	n = 293	n = 107	
**Age (years)**	69.4 ± 12.2	66.7 ± 11.9	76.9 ± 9.7	<0.001
**Male sex**	291 (72.8%)	213 (72.7%)	78 (72.9%)	0.97
**Aetiology**				0.006
Ischemic heart disease	182 (45.5%)	122 (41.6%)	60 (56.1%)	
Dilated CM	73 (18.3%)	62 (21.2%)	11 (10.3%)	
Hypertensive CM	35 (8.8%)	23 (7.8%)	12 (11.2%)	
Alcoholic CM	20 (5.0%)	18 (6.1%)	2 (1.9%)	
Drug-induced CM	14 (3.5%)	12 (4.1%)	2 (1.9%)	
Valvular disease	36 (9.9%)	25 (8.5%)	11 (10.3%)	
Hypertrophic CM	10 (2.5%)	10 (2.4%)	0 (0.0%)	
Other	30 (7.5%)	21 (7.2%)	9 (8.4%)	
**HF duration (months)**	72 (26–131)	69 (24–121)	81 (28–144)	0.08
**Mean LVEF**	41.6% ± 14.5	43.1% ± 13.9	37.5% ± 15.4	0.001
**LVEF category**				0.002
** <40%**	181 (45.3%)	118 (40.3%)	63 (58.9%)	
** 40–49%**	103 (25.7%)	81 (27.6%)	22 (20.6%)	
** ≥50%**	116 (29%)	94 (32.1%)	22 (20.6%)	
**NYHA functional class**				<0.001
I	24 (6.0%)	23 (7.8%)	1 (0.9%)	
II	251 (62.8%)	211 (72.0%)	40 (37.4%)	
III-IV	125 (31.2%)	59 (20.2%)	66 (61.7%)	
**Co-morbidities**				
Hypertension	294 (73.5%)	206 (70.3%)	88 (82.2%)	0.02
Diabetes mellitus	173 (43.3%)	122 (41.6%)	51 (47.7%)	0.28
COPD	89 (22.3%)	52 (17.7%)	37 (34.6%)	<0.001
Renal failure*	218 (54.5%)	126 (43.0%)	92 (86.0%)	<0.001
Anaemia^#^	167 (41.8%)	98 (33.4%)	69 (64.5%)	<0.001
Atrial fibrillation/flutter	170 (42.5%)	110 (37.5%)	60 (56.1%)	0.001
**Biochemical**				
Na	139 ± 3.6	139.5 ± 3.2	138.1 ± 4.2	<0.001
Haemoglobin	12.9 ± 1.7	13.2 ± 1.6	12.2 ± 1.7	<0.001
eGFR	58.3 ± 26.6	65.0± 26.4	40.2 ± 17.3	<0.001
NTproBNP	983 (311–2678)	672 (189–1573)	3436 (1617–6932)	<0.001
**Treatments**				
ACEI/ARB	331 (82.8%)	255 (87.0%)	76 (71.0%)	<0.001
Beta-blockers	359 (89.8%)	268 (91.5%)	91 (85.03%)	0.06
MRA	205 (51.3)	147 (50.2)	58 (54.2)	0.48
Loop diuretics	330 (82.5%)	224 (76.5%)	106 (99.1%)	<0.001
Digoxin	97 (24.3%)	57 (19.5%)	40 (37.46%)	<0.001
Ivabradine	37 (9.3%)	28 (9.6%)	9 (8.43%)	0.73
Statins	297 (74.3%)	217 (74.1%)	80 (74.8%)	0.89
ICD	85 (21.3%)	69 (23.5%)	16 (15.0%)	0.06
CRT	63 (15.8%)	45 (15.4%)	18 (16.8%)	0.72

Data expressed as mean ± SD, median (25th–75th percentiles) or absolute number (percentage).

*eGFR (CKD-EPI) <60 mL/min/1.73m^2^.

^#^Hb <12 g/dL in women and < 13g/dL in men.

ACEI, angiotensin-converting enzyme inhibitor; ARB, angiotensin receptor blocker; CM: cardiomyopathy; COPD, chronic obstructive pulmonary disease; CRT, cardiac resynchronization therapy; eGFR, estimated glomerular filtration rate; ICD, implantable cardioverter device; LVEF, left ventricular ejection fraction; MRA: mineral corticoid receptor antagonist; NTproBNP, N-terminal pro-brain natriuretic peptide; NYHA, New York Heart Association.

**Fig 1:**
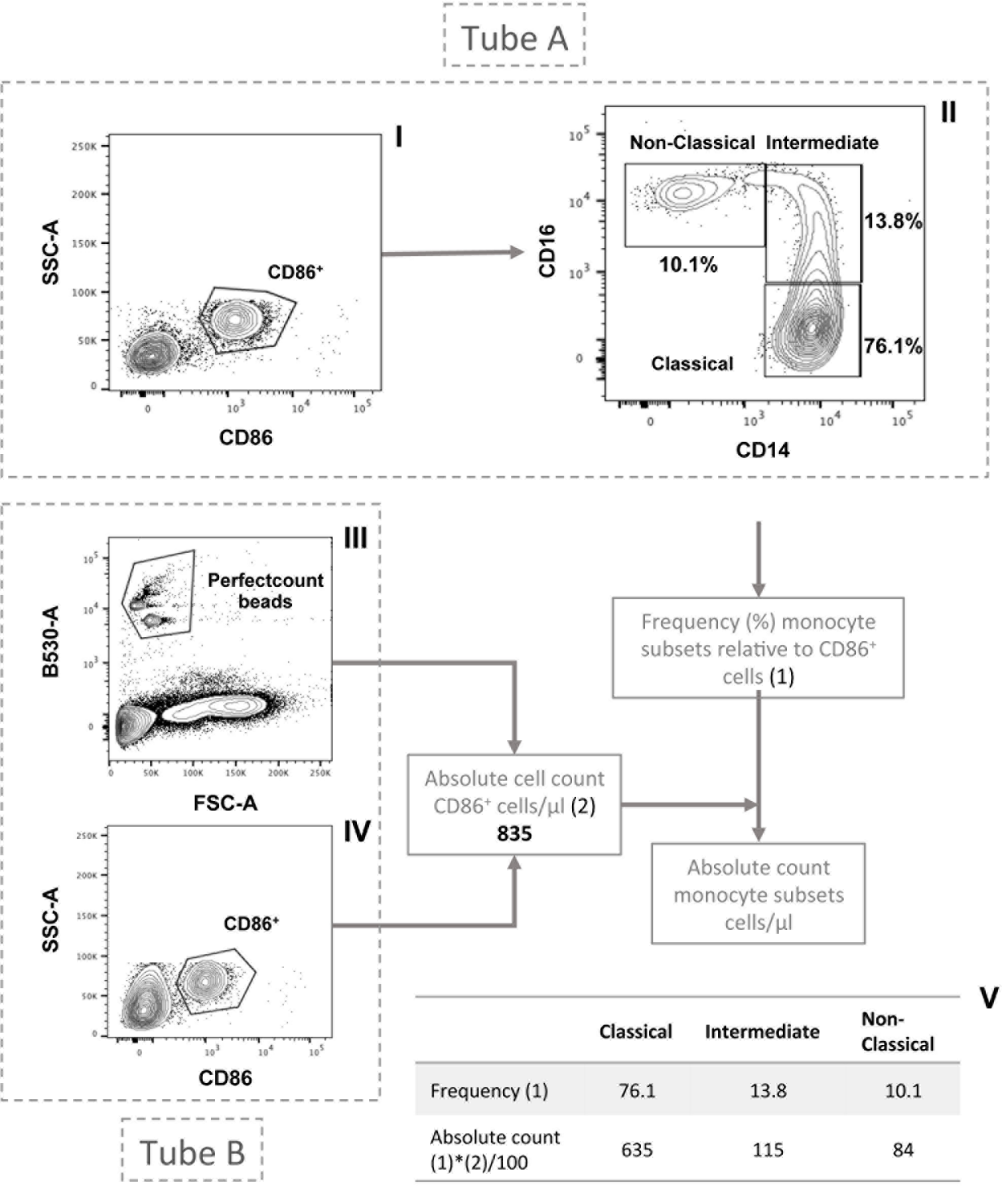
Assessment of peripheral blood monocyte subsets by flow cytometry. In tube A, total monocyte population and frequency of the monocyte subsets was defined with CD86 (**I**) and CD14/CD16 (**II**) expression, respectively. In tube B, Perfect count beads (**III**) and monocytes (CD86^+^ cells) (**IV**) were selected to determine total monocyte cell count. Frequency was then applied to total monocyte count in order to measure monocyte subsets (**V**). Table summarizes representative results from one patient with ambulatory heart failure.

Within this cohort of HF patients, we comparatively assessed three monocyte subsets—referred to as classical (CD14^++^/CD16^−^), intermediate (CD14^++^/CD16^+^), and nonclassical (CD14^+^/CD16^++^) (**Fig 1**). We did not find any correlation between subset percentage nor subset number of cells/μL and NYHA functional class, LVEF or estimated glomerular filtration rate (**S1 Table**), except for percentage of intermediate monocyte subset which showed a weak inverse correlation with LVEF (Rho –0.14, p = 0.004). When the monocyte subset distribution was assessed according to the different etiologies of HF, globally there were no statistically significant differences when percentages of subsets were considered, but a significant difference was observed in the non-classic subset when number of cells was measured (p = 0.04). In a specific comparison of such monocyte subset, valvular patients showed lower percentage (6.7 ± 3.4 *versus* 8.2 ± 4, p = 0.04) and number of cells [35.2 (23.7–63) *versus* 49.1 (34.7–70), p = 0.01] than ischemic patients and also than patients with dilated cardiomyopathy [8.3 ± 3.3, p = 0.03 and 49.7 (36.5–77.3), p = 0.006, respectively]. No differences were observed between ischemic patients and patients with dilated cardiomyopathy in any subset. No differences were also observed among etiologies in the intermediate monocyte subset.

Mean percentage and number of cells/μL of the monocyte subsets for the total cohort and for alive and deceased patients at the end of the study are summarized in Table 2. Significant differences were found between alive and deceased patients at the end of the study for the non-classical monocyte (CD14^+^/CD16^++^) subset considered as percentage, and in intermediate subset when absolute cell count (number of cells/μL) was considered (**Table 2**).

**Table 2:** Percentage and concentration of circulating monocyte subsets.

	Total	Alive	Deceased	p-value
Percentage	N = 400	N = 293	N = 107	
**CD14**^**++**^**/CD16**^**–**^	50.0 ± 17.2	50.4 ± 16.5	48.9 ± 19.08	0.45
**CD14**^**++**^**/CD16**^**+**^	42.0 ± 17.2	41.2 ± 16.5	44.0 ± 18.8	0.15
**CD14**^**+**^**/CD16**^**++**^	8.1 ± 4.0	8.42 ± 4.0	7.1 ± 4.0	0.005
**Number of cells/μL**				
**CD14**^**++**^**/CD16**^**–**^	330 (223–441)	327 (222–435)	363 (227–451)	0.38
**CD14**^**++**^**/CD16**^**+**^	258 (172–393)	253 (170–374)	303 (186–470)	0.02
**CD14**^**+**^**/CD16**^**++**^	47 (34–71)	48 (35–71)	44 (27–73)	0.10

Data expressed as mean ± SD or median (Q1-Q3).

Furthermore, Table 3 shows the univariablpoorere Cox regression analyses for risk of all-cause death, HF-related hospitalization, and the composite end-point based on percentage and absolute cell count of monocyte subsets. S2 Table shows univariable analyses for several clinical variables. When considering percentages of monocyte subsets, non-classical monocyte subset showed protective significant association with all-cause death and the composite end-point in the univariable analysis. However, in the multivariable analyses, it did not remain related any of these end-points (**S3 Table**).

**Table 3:** Univariable Cox regression analysis for risk of all-cause death, HF-related hospitalization, and the composite end-point all-cause death or HF hospitalization, based on percentage and cells/μL of monocyte subsets.

		All-cause death			HF-related hospitalization*			Composite end-point		
	HR		[95% CI]	p-value	HR	[95% CI]	p-value	HR	[95% CI]	p-value
**Percentage**										
**CD14**^**++**^**/CD16**^**–**^	1.00		[0.98–1.01]	0.38	1.00	[0.98–1.01]	0.52	1.00	[0.99–1.01]	0.53
**CD14**^**++**^**/CD16**^**+**^	1.01		[1.00–1.02]	0.12	1.00	[0.99–1.01]	0.44	1.01	[1.00–1.02]	0.23
**CD14**^**+**^**/CD16**^**++**^	0.93		[0.88–0.98]	0.004	0.99	[0.94–1.04]	0.66	0.95	[0.91–0.99]	0.02
**Number of cells/**μ**L**^#^										
**CD14**^**++**^**/CD16**^**–**^	1.07		[0.88–1.31]	0.49	1.04	[0.84–1.29]	0.71	1.05	[0.90–1.24]	0.54
**CD14**^**++**^**/CD16**^**+**^	1.29		[1.06–1.56]	0.01	1.18	[0.99–1.41]	0.07	1.18	[1.01–1.38]	0.04
**CD14**^**+**^**/CD16**^**++**^	0.83		[0.70–0.99]	0.04	1.00	[0.81–1.23]	0.99	0.87	[0.75–1.01]	0.08

*Death has been considered as competitive risk for HF-related hospitalization.

#Log-transformed and per 1 SD.

**Fig 2:**
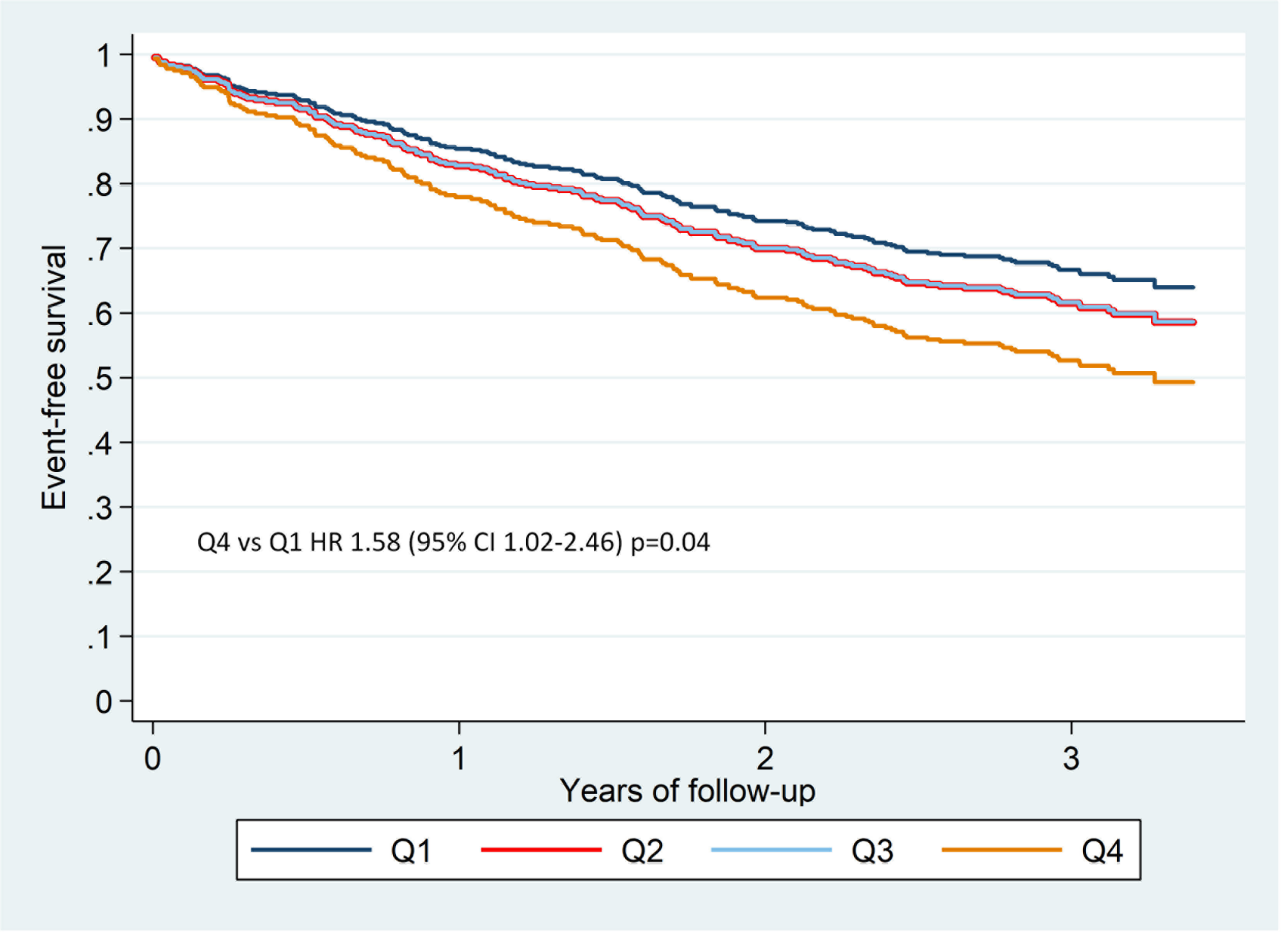
Event-free survival curves for the composite end-point of all-cause death or HF-related hospitalization, relative to quartiles of number of cells/μL of the intermediate (CD14^++^/CD16^+^) monocyte subset. HR Q4 *versus* Q1: 1.58 (95% CI 1.02–2.46), p = 0.04).

**Fig 3:**
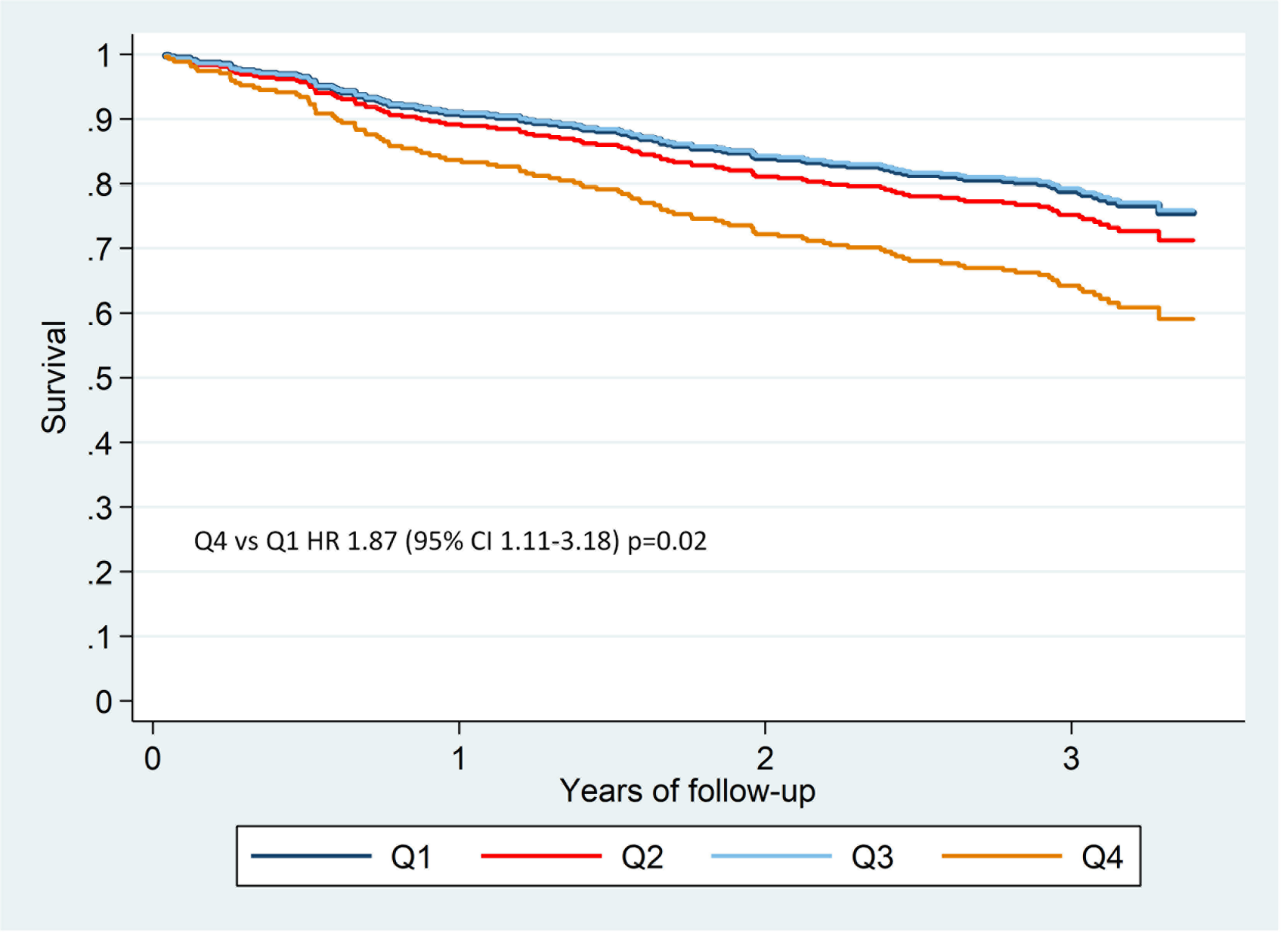
Survival curves for all-cause death, relative to quartiles of number of cells/μL of the intermediate (CD14^++^/CD16^+^) monocyte subset. HR Q4 *versus* Q1: 1.87 (95% CI 1.11–3.18), p = 0.02.

By contrast, when considering absolute cell count, the intermediate (CD14^++^/CD16^+^) monocyte subset showed detrimental association with all-cause death and the composite end-point and showed borderline significance with HF-related hospitalization, while the non-classical (CD14^+^/CD16^++^) subset showed protective association with all-cause death and borderline significance with the composite end-point in the univariable analyses (**Table 3**). Fig 2 depicts free-event survival curves for quartiles (number of cells/μL) for CD14^++^/CD16^+^ monocytes. The fourth quartiles showed a 58% increase in the risk of suffering a composite end-point relative to the first quartile (p = 0.04), and Fig 3 depicts survival curves for the same quartiles. Fourth quartile showed an 87% increase in the risk of all-cause death relative to first quartile (p = 0.02). In multivariable analyses, however, only the intermediate monocyte subset remained associated with all-cause death (p = 0.03) and with the composite end-point (p = 0.02) (**Table 4**).

**Table 4:** Multivariable Cox regression analysis for risk of all-cause death, HF-related hospitalization, and the composite end-point all-cause death or HF hospitalization, including number of cells/μL of CD14^++^CD16^+^ (intermediate) and CD14^+^/CD16^++^ (non-classic) monocyte subsets when appropriate.

	All-cause death			HF-related hospitalization*			Composite end-point		
	HR	[95% CI]	p-value	HR	[95% CI]	p-value	HR	[95% CI]	p-value
**CD14**^**++**^**/CD16**^**+**^, ^#^	1.25	[1.02–1.52]	0.03	—	—	—	1.20	[1.03–1.40]	0.02
**CD14**^**+**^**/CD16**^**++**^, ^#^	—	—	—				—	—	—
**Age**	1.04	[1.02–1.06]	<0.001	—	—	—	1.02	[1.00–1.03]	<0.05
**Female sex**	—	—	—	—	—	—	—	—	—
**NYHA functional class**	2.17	[1.45–3.25]	<0.001	—	—	—	2.21	[1.60–3.06]	<0.001
**LVEF**	—	—	—	—	—	—	—	—	—
**Haemoglobin**	—	—	—	—	—	—	—	—	—
**Sodium**	0.94	[0.89–0.99]	0.01	—	—	—	—	—	—
**eGFR**	—	—	—	—	—	—	—	—	—
**NTproBNP**^#^	2.69	[2.06–3.51]	<0.001	2.10	[1.68–2.61]	<0.001	2.17	[1.78–2.66]	<0.001

* Death has been considered as competitive risk for HF-related hospitalization

# Log-Transformed and per 1 SD. eGFR, estimated glomerular filtration rate

HF, heart failure; LVEF, left ventricular ejection fraction; NTproBNP, N-terminal pro-brain natriuretic peptide; NYHA, New York Heart Association.

## Discussion

Monocytes can have both damage and repair functions in different cardiovascular diseases, depending on the subset. In healthy individuals, monocyte subset distribution and function showed a significant prevalence of classical (CD14^++^/CD16^-^) monocyte subset (80.1±7%) in comparison to the other two subsets defined as intermediate (CD14^++^/CD16^+^; 3.7±2.0%) and non-classical (CD14^+^/CD16^++^; 6.2±2.8%) [20]. Different distributions of monocyte subsets in disease contexts may be related to pathogenesis and the specific functions of classical, intermediate, and non-classical monocytes [21].

We investigated two main questions in this study: first, what is the distribution of the three subsets of monocytes in outpatients with HF diagnosis, and second, can this distribution be related to the main events of the study, and therefore play predictive role for HF patients? In this context, we found a significant difference in the percentages of monocyte subsets relative to previous studies performed in healthy controls [20]. The predominant monocyte subset was the classical (50.0±17.2%), followed by the intermediate (42±17.2%) subset; the non-classical subset of monocytes occurred with much less frequency (8.1±4.0%). This represents a significant expansion of the intermediate subset in HF in comparison with healthy controls.

The CD14^+^/CD16^+^ intermediate subset is not yet completely understood. It has been reported to have characteristics similar to both the classical and non-classical monocytes (high phagocytic activity, antigen presentation and T-cell interaction [22–24]), and some studies have suggested that it may represent a stage of differentiation to immature classical and non-classical subsets [23]. The intermediate subset has been associated with many clinical conditions, ranging from chronic inflammation and type 2 diabetes mellitus to chronic vascular and endothelial damage and atherosclerosis [14, 25, 26]. Intermediate monocytes have a more pro-inflammatory capacity than the non-classical subset [27], as they secrete more oxygen radicals, TNF-α, and IL-1β [26]. Collectively, this may explain the elevated levels of intermediate monocytes in our HF cohort, in which the majority of the patients were men with chronic HF with ischemic aetiology. For instance, the intermediate subset experienced a significant dynamic elevation of its blood levels in ST-elevation myocardial infarction, which correlated with troponin elevation and left ventricular function [24]. To our knowledge, there is only one study reporting the distribution of monocytes in patients with acute and stable ischemic HF [10], but conclusive evidence of the significance and importance of the monocyte distribution in patients with chronic HF is still not yet well understood. The intermediate subset was increased in patients with both acute and stable HF in the previous studies [28], as seen in our cohort in comparison to healthy individuals. In this context, the maintenance of cardiac integrity through removal of irreparably dead cells is crucial. An influx of pro-inflammatory cells such as intermediate monocytes into the damaged area is thought to be essential for the very early wound healing process, but their persistence beyond the initial repair phase could extend longer-term inflammation-related adverse effects into healthy remote myocardial areas. This could be a potential mechanistic explanation of the poorer prognosis of HF patients with higher intermediate monocytes.

A significant difference was found for the intermediate subsets when number of cells/μL was addressed. Remarkably, although patients who died had worse clinical characteristics (higher age, worse NYHA functional class, worse LVEF, higher NTproBNP, worse renal function) the intermediate subset was independently associated with all-cause death and the composite end-point in the multivariable analyses. Wrigley *et al*. previously reported that the expanded intermediate subset was associated with poorer prognosis in patients with acute HF [10]. It should be emphasized that our cohort is an ambulatory chronic cohort, with much less inflammation activation than seen in acute HF, but we also found such prognostic relationship. Other studies have indicated that the intermediate subset proved to be risk factor for post-ST elevation myocardial infarction adverse outcomes and cardiovascular events [29, 30]. Also, the amount of intermediate monocytes was found to correlate with worse cardiac function and predicted the possibility to reach an improvement in NYHA functional class at 3 months after transcatheter aortic valve replacement [31].

An additional relevant finding in this study is the assessment and prognostic value of monocyte subset absolute cell count, beyond the classical approach of subset percentages. Monocyte cell count (defined as number of cells/μL) has been assessed in previous studies of healthy patients [20]. We found that the absolute count was markedly superior to the percentage distribution of each subset when considering the prognostic role of the different monocyte subsets. An increase or decrease in subpopulation percentage may reflect a change in another cell subtype in addition to the subset of interest, and this issue could be resolved by using absolute values combined with relative values. Absolute cell counts have been shown to be a major tool for laboratory diagnosis for a variety of pathological conditions, including CD4^+^ T-cells for Acquired ImmunoDeficiency Syndrome and haematopoietic progenitor cells expressing CD34 for cord blood transplants and autotransplantation [32, 33]. T-cell count imbalances have also been described in association with protein-caloric malnutrition, anorexia nervosa, lactation and iron deficiency, autoimmune thyroiditis, bronchial asthma, multiple sclerosis, polymyalgia rheumatica, and rheumatoid arthritis [34].

The possibility of selection bias represents a potential limitation of the study. The participants reported here were drawn from a general population that visited our tertiary hospital HF Clinic. The majority had been admitted to the hospital in previous years and the cohort included primarily male patients with ischemic heart disease as the main cause of HF. We have analyzed only one blood sample per patient, and cannot comment on the prognostic value of serial determinations. Indeed, although blood samples were obtained in routine ambulatory visits, we cannot discard that in some isolated patient it could have been obtained after a relatively near inciting incident (i.e. exacerbation) which could have mobilized the cells of interest. We did not have data on other inflammatory cytokines such as TNF, IL6, or IL1b, which might could yield insights into the mechanistic role of differential monocyte distribution.

## Conclusion

This investigation evaluated the relationship between circulating monocyte subsets and HF. The classical (CD14^++^/CD16^–^) monocyte subset was decreased and the intermediate (CD14^++^/CD16^+^) monocyte subset was increased in patients with HF compared to reported controls. The quantification of the absolute cell count of each monocyte subset (number of cells/μL) showed a superior prognostic value for the studied cohort of patients, compared to the performance of monocyte subset analysis by percentages. Moreover, the intermediate subset was independently associated with all-cause death and the composite end-point of all-cause death or HF hospitalization, in multivariable analyses. Indeed, further studies are necessary to confirm our preliminary findings and to better underscore the clinical value of these data.

## Supporting information

S1 Table Correlations among monocyte subsets and clinical variables. (DOCX)

S2 Table Univariable Cox regression analysis for risk of all-cause death, HF-related hospitalization, and the composite end-point all-cause death or HF-related hospitalization based on clinical variables. (DOCX)

S3 Table Multivariable Cox regression analysis for risk of all-cause death, HF-related hospitalization, and the composite end-point all-cause death or HF hospitalization, including percentage of the CD14^+^/CD16^++^ (non-classic) monocyte subset. (DOCX)
