# Supplementary material for: Circulating monocyte subsets and heart failure prognosis
Source: PLoS One. 2018 Sep 21;13(9):e0204074. doi: 10.1371/journal.pone.0204074 (PMC6150659; doi:10.1371/journal.pone.0204074)
Supplement: S1 Table — (DOCX) [file pone.0204074.s001.docx]

**S1 Table.** Correlations among monocyte subsets and clinical variables

|  | **NYHA** | **LVEF** | **eGFR** |
| --- | --- | --- | --- |
| **Percentage*** |  |  |  |
| CD14^++^/CD16^–^ | r= –0.03, p=0.54 | r= 0.01, p=0.80 | r= 0.05, p=0.30 |
| CD14^++^/CD16^+^ | r= 0.04, p=0.38 | r= –0.08, p=0.11 | r= –0.07, p=0.18 |
| CD14^+^/CD16^++^ | r= –0.05, p=0.29 | r= 0.05, p=0.33 | r= 0.06, p=0.21 |
| **Number of cells/µL^#^** |  |  |  |
| CD14^++^/CD16^–^ | rho= –0.04, p=0.38 | rho= –0.03, p=0.54 | rho= 0.03, p=0.55 |
| CD14^++^/CD16^+^ | rho= 0.03, p=0.50 | rho= –0.14, p=0.004 | rho= –0.09, p=0.09 |
| CD14^+^/CD16^++^ | rho= –0.07, p=0.20 | rho= –0.01, p=0.93 | rho= 0.04, p=0.40 |

*Correlations assessed by Pearson correlation coefficient. #Correlations assessed by Spearman’s rank correlation coefficient (rho); NYHA, New York Heart Association; LVEF, left ventricular ejection fraction; eGFR, estimated glomerular filtration rate.
