# Supplementary material for: Circulating monocyte subsets and heart failure prognosis
Source: PLoS One. 2018 Sep 21;13(9):e0204074. doi: 10.1371/journal.pone.0204074 (PMC6150659; doi:10.1371/journal.pone.0204074)
Supplement: S2 Table — (DOCX) [file pone.0204074.s002.docx]

**S2 Table.** Univariable Cox regression analysis for risk of all-cause death, HF-related hospitalization, and the composite end-point all-cause death or HF-related hospitalization based on clinical variables.

|  | **All-cause death** | | | **HF-related hospitalization*** | | | **Composite end-point** | | |
| --- | --- | --- | --- | --- | --- | --- | --- | --- | --- |
|  | **HR** | **[95% CI]** | **p-value** | **HR** | **[95% CI]** | **p-value** | **HR** | **[95% CI]** | **p-value** |
| **Age** | 1.08 | [1.06-1.10] | <0.001 | 1.02 | [1.00-1.04] | 0.03 | 1.05 | [1.04-1.07] | <0.001 |
| **Female sex** | 0.99 | [0.64-1.51] | 0.94 | 0.79 | [0.49-1.26] | 0.32 | 0.99 | [0.60-1.40] | 0.94 |
| **Ischemic aetiology** | 1.56 | [1.07-2.29] | 0.02 | 1.26 | [0.85-1.87] | 0.25 | 1.23 | [0.90-1.68] | 0.19 |
| **NYHA functional class** | 4.75 | [3.27-6.89] | <0.001 | 2.15 | [1.54-2.98] | <0.001 | 3.92 | [2.92-5.26] | <0.001 |
| **LVEF** | 0.97 | [0.96-0.99] | <0.001 | 0.99 | [0.97-1.00] | 0.09 | 0.98 | [0.97-0.99] | 0.002 |
| **Haemoglobin** | 0.70 | [0.62-0.79] | <0.001 | 0.80 | [0.71-0.90] | <0.001 | 0.72 | [0.65-0.79] | <0.001 |
| **Na** | 0.91 | [0.86-0.95] | <0.001 | 0.98 | [0.92-1.03] | 0.38 | 0.93 | [0.89-0.97] | <0.001 |
| **eGFR** | 0.97 | [0.96-0.97] | <0.001 | 0.98 | [0.97-0.99] | <0.001 | 0.97 | [0.97-0.98] | <0.001 |
| **NTproBNP^#^** | 3.15 | [2.53-3.93] | <0.001 | 2.10 | [1.68-2.61] | <0.001 | 2.74 | [2.30-3.27] | <0.001 |

*For HF-related hospitalization death has been taken into account as competitive risk. #Log-transformed and per 1 SD. eGFR, estimated glomerular filtration rate; HF, heart failure; LVEF, left ventricular ejection fraction; NTproBNP, N-terminal pro-brain natriuretic peptide; NYHA, New York Heart Association.
