# Supplementary material for: Circulating monocyte subsets and heart failure prognosis
Source: PLoS One. 2018 Sep 21;13(9):e0204074. doi: 10.1371/journal.pone.0204074 (PMC6150659; doi:10.1371/journal.pone.0204074)
Supplement: S3 Table — (DOCX) [file pone.0204074.s003.docx]

**S3 Table.** Multivariable Cox regression analysis for risk of all-cause death, HF-related hospitalization, and the composite end-point all-cause death or HF hospitalization, including percentage of the CD14^+^/CD16^++^ (non-classic) monocyte subset.

|  | **All-cause death** | | | **Composite end-point** | | |
| --- | --- | --- | --- | --- | --- | --- |
|  | **HR** | **[95% CI]** | **p-value** | **HR** | **[95% CI]** | **p-value** |
| **Percentage CD14^+^/CD16^++^** | -- | --- | -- | -- | --- | -- |
| **Age** | 1.04 | [1.02-1.06] | <0.001 | 1.02 | [1.00-1.03] | 0.03 |
| **Female sex** | -- | --- | -- | -- | --- | -- |
| **Ischemic aetiology** | -- | --- | -- |  |  |  |
| **NYHA functional class** | 2.07 | [1.39-3.09] | <0.001 | 1.97 | [1.42-2.73] | <0.001 |
| **LVEF** | -- | --- | -- | -- | --- | -- |
| **Haemoglobin** | -- | --- | -- | -- | --- | -- |
| **Sodium** | 0.93 | [0.88-0.97] | 0.002 | 0.93 | [0.92-1.00] | 0.03 |
| **eGFR** | -- | --- | -- | -- | --- | -- |
| **NTproBNP^#^** | 2.71 | [2.08-3.52] | <0.001 | 2.23 | [1.82-2.74] | <0.001 |

#Log-Transformed and per 1 SD. eGFR, estimated glomerular filtration rate; HF, heart failure; LVEF, left ventricular ejection fraction; NTproBNP, N-terminal pro-brain natriuretic peptide; NYHA, New York Heart Association.
